# Mutant *gltS* alleles enable a *Vibrio fischeri* D-glutamate auxotroph to grow with lower requirements for exogenous D-glutamate

**DOI:** 10.1128/spectrum.01025-25

**Published:** 2025-09-04

**Authors:** Macey Coppinger, Richard F. Helm, Liu Yang, Edward G. Ruby, David L. Popham, Eric V. Stabb

**Affiliations:** 1 Department of Microbiology, University of Georgia 1355, Athens, Georgia, USA; 2 Department of Biological Sciences, University of Illinois at Chicago, Chicago, Illinois, USA; 3 Department of Biochemistry, Virginia Tech 1757, Blacksburg, Virginia, USA; 4 Division of Biosphere Sciences and Engineering, California Institute of Technology 6469, Pasadena, California, USA; 5 Department of Biological Sciences, Virginia Tech 1757, Blacksburg, Virginia, USA; Rensselaer Polytechnic Institute, Troy, New York, USA

**Keywords:** photobacterium, *Aliivibrio*, peptidoglycan, D-amino acids, glutamate, transport

## Abstract

D-glu is a key component of peptidoglycan (PG) and is essential for growth in most bacteria. To assess constraints on PG evolution and bacterial requirements for D-glu, we sought to artificially evolve PG biosynthesis, leading to either replacement of D-glu in the PG peptide or alternative pathways to D-glu incorporation. We previously found that suppression of D-glu auxotrophy in a *murI racD* mutant of *Vibrio fischeri* grown on lysogeny broth salts (LBS) medium was rare but could be accomplished by mutation of *bsrF*, with restoration of wild-type PG structure. Here, we selected nine additional prototrophic suppressors of the same *murI racD* mutant from 10^10^ colony-forming units (CFU) plated on LBS supplemented with ~2.7 mM D-gln. Each suppressor had a mutation in *gltS*, which encodes a putative sodium:glutamate symporter. Increased copy numbers of mutant *gltS* alleles enabled growth on unsupplemented LBS and resulted in PG containing D-glu. Examination of media components suggests that D-gln supplementation had inadvertently added ~14 μM D-glu, and that LBS itself contains ~1.4 μM D-glu. The mutations in *gltS* enabled growth with similarly low D-glu concentrations, but also increased sensitivity to homocysteic acid, suggesting more promiscuous transport. Surprisingly, we discovered that expression of mutant *gltS* in the auxotroph leads to incorporation of lysine into PG, in addition to canonical D-glu. When seawater is supplemented with D-glu, this *V. fischeri* mutant still colonized *Euprymna scolopes* and triggered PG-induced morphogenesis. Our results shed light on glutamate transport, highlight trade-offs in GltS structure and function, and reveal an unusual PG modification.

**IMPORTANCE:**

D-glu is an important building block in the peptidoglycan (PG) component of the bacterial cell wall, and its endogenous production is considered essential in most bacteria, even when grown in complex media. In *Vibrio fischeri*, *in trans* expression of mutant GltS symporters allows D-glu auxotrophic strains to grow on lysogeny broth salts (LBS) medium without exogenous D-glu, although there is a fitness trade-off of increased sensitivity to homocysteic acid. Our finding that LBS contains sufficient D-glu to support robust growth highlights the undervalued importance of D-amino acid transport and the ubiquity of D-amino acids. Moreover, the discovery of D-lysine in the PG peptide is an unusual PG modification that warrants further study.

## INTRODUCTION

D-amino acids play important roles in biology and are widespread in the biosphere. Although less abundant than proteinogenic L-amino acids, D-amino acids can be formed enzymatically or abiotically from their enantiomeric L-amino acid counterparts (1–3), and they serve nutritional and other functional roles for bacteria (4–9). D-ala and D-glu play well-known roles for bacteria as highly conserved components of the peptidoglycan (PG) cell wall (10, 11). In this context, the presence of D-amino acids in the PG side chain may render PG more resistant to proteases, which typically target L-L peptide bonds (12). The bacteria-specific structure of PG underlies its targeting as a microbe-associated molecular pattern recognized by bacteria-surveillance systems of plants and animals with the peptide chain being an important recognition determinant (13, 14). PG and its fragments play important roles in symbiont recognition in the mutualism between the Hawaiian bobtail squid *Euprymna scolopes* and *Vibrio fischeri* (15), which prompted our interest in PG structure and biosynthesis in this bacterium. We have used *V. fischeri* as a model for exploring the experimentally forced evolution of new PG biosynthetic pathways, providing insight into PG structural variation, as well as the constraints on that variation. Our strategy has been to block PG biosynthesis and select for suppressor mutants that can grow without a gene normally considered essential. In this and in an earlier study (16), we focused on the D-glu moiety of PG.

D-glu is a critical component of the PG for most bacteria and is supplied for PG biosynthesis by the activity of glutamate racemase(s), usually encoded by a *murI* (*racE*) gene (17–19), which has been categorized as essential to many bacteria (20–22). In general, “essential” in these experimental contexts means the gene is required to grow in a rich medium such as lysogeny broth (LB), which provides a complex source of nutrients that can obviate the need for many endogenous biosynthetic pathways. In some bacteria, *murI* mutants can be obtained on media supplemented with D-glu (23–25), though in *Escherichia coli,* recovery of a *murI* mutant on LB required both addition of D-glu and secondary mutations in *gltS*, which encodes a glutamate transporter (26, 27).

As in many other bacteria, *murI* was categorized as an essential gene in *V. fischeri*, in this case based on an InSeq analysis (28). A *murI*::Tn mutant was subsequently recovered on LB salts (LBS) supplemented with ~2.7 mM D-glu (24). Previously, we found that growth on unsupplemented LBS could be restored to a *V. fischeri murI* mutant either by the overexpression of the aspartate racemase RacD (29) or by removal of a secretion signal sequence from the broad-spectrum racemase BsrF (16). No other suppressors of D-glu auxotrophy in the *murI racD* mutant were recovered on LBS, despite plating >10^10^ cells (16). One goal of those experiments was to determine if rare mutations could result in replacement of the D-glu moiety of PG, and toward that end, we selected for suppressors of D-glu auxotrophy in media supplemented with D-gln or iso-D-gln (16). D-gln and iso-D-gln were used in this context due to their structural similarity to D-glu. We reasoned that mutants might either substitute these D-amino acids or their derivatives into the PG, or they may be converted into D-glu to generate canonical PG. Here, we report the results of our attempts to select prototrophic suppressors of the *murI racD* mutant on LBS supplemented with ~2.7 mM D-gln.

## RESULTS

### Mutations in *gltS* allow a D-glu auxotroph to grow with relatively low concentrations of D-glu

**Fig 1:**
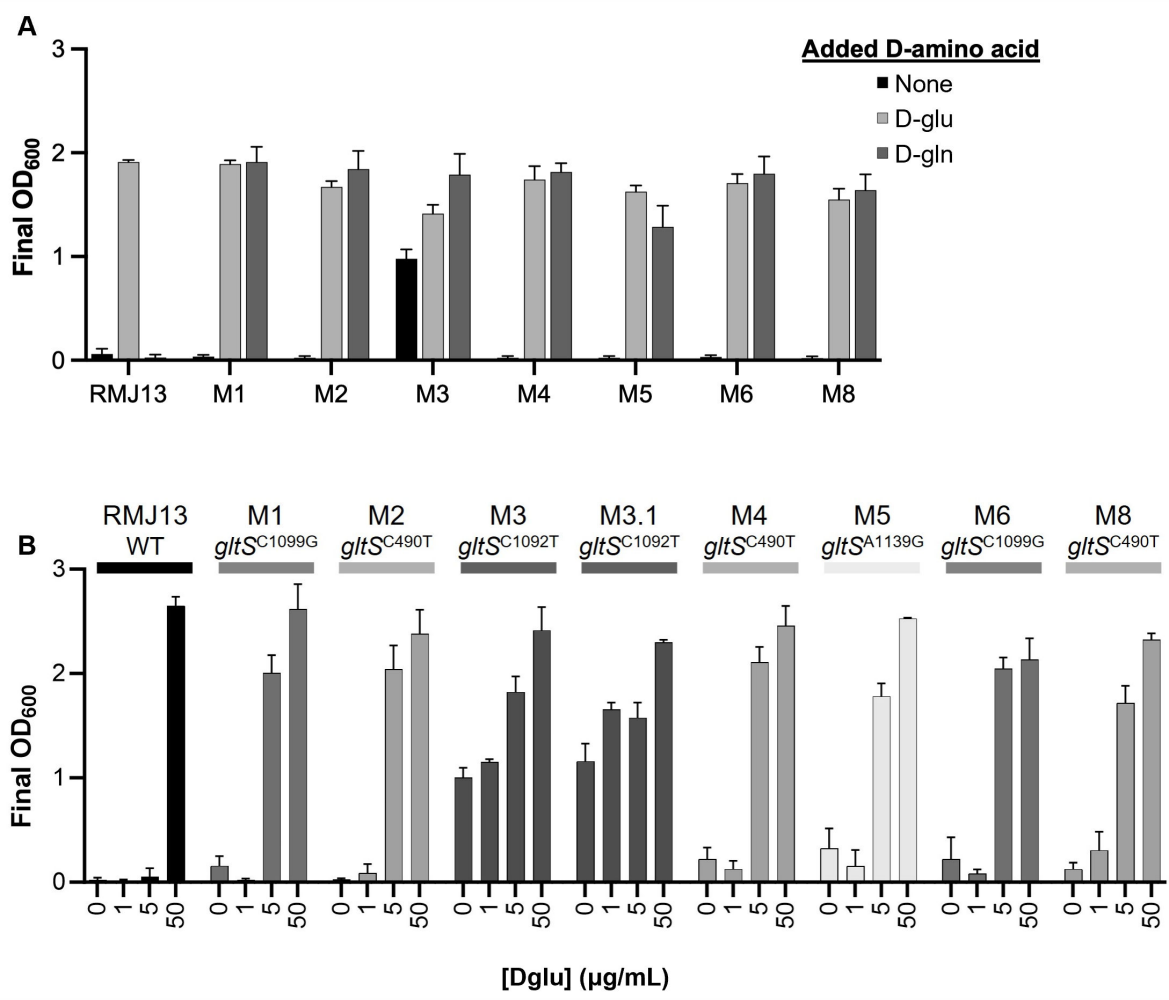
Growth of RMJ13 and suppressor mutants with and without D-glu or D-gln supplementation. (A) Final OD_600_ of RMJ13 (*murI*::mini-Tn*5* ∆*racD*) and its derivatives RMJ13M1 through RMJ13M8 (denoted here as M1 through M8) grown in LBS-Em supplemented with 400 µg/mL D-gln or D-glu. Stocks of D-gln were subsequently found to contain small amounts of D-glu, as described below. (B) D-glu requirements for *gltS* mutants were assessed by growing strains in LBS-Em containing 0, 1, 5, or 50 µg/mL D-glu supplementation. Cultures were grown for 24 hours before reading OD_600_. Error bars indicate standard error of the mean (*n* = 4). Data from one representative experiment of at least three are shown.

We attempted to select for mutants of RMJ13 (∆*racD murI*::mini-Tn*5*-Em) able to grow without D-glu supplementation by plating cells on LBS containing erythromycin (LBS-Em) further supplemented with ~2.7 mM (400 µg/mL) D-gln. Erythromycin was included in the plates because we previously found that the mini-Tn*5*-Em could precisely delete from *murI*, leading to prototrophic *murI* revertants (16). After plating over 10^10^ colony-forming units (CFU) total, we isolated nine suppressor mutants, RMJ13M1 through RMJ13M9, yielding a recovery rate of 7 × 10^−10^. At least seven of these nine mutants arose independently, while two may be siblings, as described below. These strains grew when LBS-Em was supplemented with 400 µg/mL exogenous D-glu or D-gln but, with one exception, did not grow in unsupplemented LBS-Em (Fig. 1A). The exception was mutant RMJ13M3, which displayed an inconsistent requirement for supplementation and sometimes grew on unsupplemented LBS-Em. While working with RMJ13M3, we isolated derivative RMJ13M3.1, which consistently grew on unsupplemented LBS-Em.

Sequencing revealed that RMJ13M1 through RMJ13M9 each contain a mutation in *gltS* (VF_A0507) (Table 1), which encodes a putative sodium:glutamate symporter (30, 31). For most strains, these *gltS* alleles were revealed by whole-genome sequencing, while RMJ13M7 and RMJ13M9 *gltS* alleles were targeted directly for cloning and sequencing. Four different mutant *gltS* alleles were recovered (Table 1): C490T, C1092T, C1099G, and A1139G. Mutations C490T and C1099G were isolated from multiple independent cultures, while C1092T and A1139G each arose only once. Although some of these strains also have additional mutations, the one commonality was mutation of *gltS* (Table 1). Mutant RMJ13M7 has the same *gltS* allele as RMJ13M6 and was isolated from the same culture; and mutant RMJ13M9 was isolated from the same culture as RMJ13M8 and has the same *gltS* allele. Thus, RMJ13M7 and RMJ13M9 could be clonal siblings to RMJ13M6 and RMJ13M8, respectively, and are not further analyzed in this study.

**TABLE 1:** List of RMJ13-derivative strains and their *gltS* alleles

RMJ13 mutant	*gltS* allele	Other mutations
RMJ13.M1	*gltS* ^C1099G^	None
RMJ13.M2	*gltS* ^C490T^	VF_0468^A699G^*gacS*^G644C^
RMJ13.M3	*gltS*^C1092T^ Duplication	Amplification junction [ACTTAACTTGAT::GATGTTGTTTTA]
RMJ13.M3.1	*gltS*^C1092T^ ~10× amplification	VF_2147^G778A^ Amplification junction [ACTTAACTTGAT::GATGTTGTTTTA]
RMJ13.M4	*gltS* ^C490T^	*gacS* ^G644C^
RMJ13.M5	*gltS* ^A1139G^	None
RMJ13.M6	*gltS* ^C1099G^	VF_0468^A699G^
RMJ13.M7	*gltS* ^C1099G^	Unknown
RMJ13.M8	*gltS*^C490T^ Duplication	Duplication junction [TGTGCTGATAAA::AGGTGAAAAGGG]
RMJ13.M9	*gltS* ^C490T^	Unknown

After recovering these suppressor mutants, we subsequently discovered that our D-gln stocks contain low amounts of D-glu. Though the stocks were prepared to contain ~273 mM D-gln, measurements indicated the stocks had ~10.4 mM D-gln and ~1.4 mM D-glu, likely due to unexpected degradation and spontaneous deamidation of D-gln (32) (Table 2). This finding led us to speculate that mutant GltS symporters are less likely to be promiscuously transporting D-gln, but more likely have increased efficiency of D-glu transport. To test this hypothesis, we grew RMJ13 as well as eight of the derivative mutants on LBS-Em with varying concentrations of exogenous D-glu. The parental auxotroph required a minimum of 111 µM (16 µg/mL) exogenous D-glu in LBS-Em medium. By contrast, the derivative strains require less D-glu, growing consistently in LBS-Em supplemented with just 5 µg/nL (Fig. 1B). Suppressor strains RMJ13M3 and RMJ13M3.1 do not require any D-glu supplementation in LBS-Em.

**TABLE 2:** Average D-amino acid concentrations in various media and solutions^*a*,*b*^

	Concentration (μM)			
	L-glu	D-glu	L-gln	D-gln
LBS	93.57 ± 9.28	1.41 ± 0.07	–	–
YEBS^*c*^	185.33 ± 45.15	1.70 ± 0.21	0.73 ± 0.05	–
TBS^*d*^	60.06 ± 0.37	–	0.68 ± 0.03	–
D-glu stock	1,105 ± 0.7	89,400 ± 654	–	–
D-gln stock	9.2 ± 0.89	1,488 ± 27	–	10,447 ± 118

^*a*^ “–“ indicates that amino acid was not detected.

^*b*^ Experiment was performed twice, each run in triplicate. Results from one experiment are shown as average ± standard deviation (*n *= 3).

^*c*^ YEBS, yeast extract broth saline.

^*d*^ TBS, tryptone broth saline.

### Increased copy number amplifies phenotypes of mutant *gltS* alleles

Based on the analysis of shotgun sequencing depth, RMJ13M3, RMJ13M3.1, and RMJ13M8, which contain *gltS* alleles C490T (RMJ13M3 and RMJ13M3.1) and C1092T (RMJ13M8), appear to have amplifications of large regions of chromosome II that include *gltS*. Based on the sequencing depth and identification of a novel chromosomal junction (Table 1), RMJ13M8 has a duplication of an 83 kb region that includes the C1092T mutant *gltS* allele. Strains RMJ13M3 and RMJ13M3.1 share an identical amplification junction (Table 1), with an amplified region of about 60 kb that includes the mutant allele of *gltS*. As noted above, RMJ13M3 has an inconsistent requirement for amino acid supplementation, which may be due to spontaneous amplification and resolution of the chromosomal duplication, leading to varied *gltS* copy number and, thus, the amount of GltS expressed in the cells. RMJ13M3.1 was isolated on LBS-Em without supplementation and consistently grew without additional supplementation. Based on sequencing depth, RMJ13M3.1 has approximately 10 copies of the amplified region encompassing *gltS* (Table 1). These data led us to hypothesize that increased copy number of mutant *gltS* alleles leads to increased D-glu transport, thereby decreasing the amount of exogenous D-glu needed to support growth of these auxotrophic strains.

**Fig 2:**
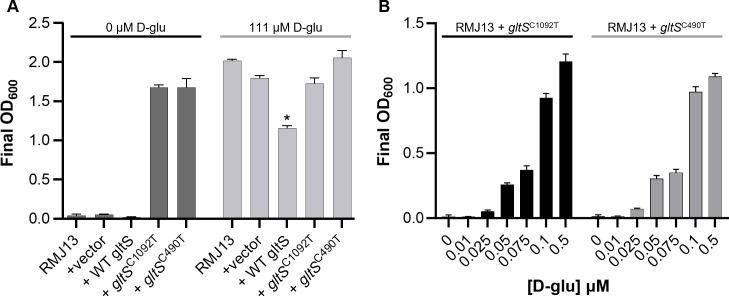
Mutant *gltS* alleles lower D-glu requirement of D-glu auxotroph. (A) Final OD_600_ readings for *V. fischeri* cultures grown for 24 hours in LBS-Em with or without supplementation of 50 µg/mL D-glu. Strains included RMJ13 alone or carrying vector pVSV105, pMNC15 (*gltS*), pMNC16 (*gltS*^C1092T^), or pMNC17 (*gltS*^C490T^). (B) Final OD_600_ readings for *V. fischeri* cultures grown for 24 hours in *fischeri* minimal medium with varying amounts of D-glu as indicated. Error bars indicate standard error of the mean (*n* = 3). Data from one representative experiment of at least three are shown. * RMJ13 expressing WT GltS (carried by pMNC15) grows to a significantly lower final OD_600_ than all other strains (*P* < 0.05, Student’s *t*-test).

To test this hypothesis, we cloned wild-type *gltS* as well as two of the mutant *gltS* alleles (C490T and C1092T) into shuttle vector pVSV105 and moved them into RMJ13. It was estimated previously that *V. fischeri* holds 10 to 15 copies of pVSV105 per cell on average (33). *In trans* expression of mutant *gltS* alleles on this shuttle vector enabled growth of RMJ13 on LBS-Em without supplementation, while the wild-type *gltS* and the parental vector did not (Fig. 2A). These results, in combination with data of the mutant strains, are consistent with our hypothesis that higher expression of these mutant forms of GltS improves cells’ ability to suppress D-glu auxotrophy by enabling them to access external D-glu at lower concentrations.

### LBS contains trace amounts of D-glu

The observation that mutant *gltS* alleles could enable growth on lower exogenous levels of D-glu still begs the question of how they support growth on LBS. The nutrient-rich components of LBS, tryptone and yeast extract, are known to contain L-amino acids, but to our knowledge, the presence of D-amino acids has not been reported or quantified. Based on our results, we predicted that LBS does contain D-amino acids, though likely in concentrations that are not easily measured or utilized by bacteria. We analyzed LBS, as well as less-complex media derivatives tryptone broth saline (TBS) and yeast extract broth saline (YEBS) via solid phase extraction and mass spectrometry, and the results of one trial are listed in Table 2. Importantly, these data indicate that LBS contains ~1.4 µM (0.2 µg/mL) D-glu, mostly supplied by the yeast extract (Table 2). When grown in defined *fischeri* minimal medium (FMM) (16, 34), expression of *gltS*^C490T^ or *gltS*^C1092T^ can support growth in similarly low, submicromolar concentrations of D-glu, although the strains do still require D-glu (Fig. 2B).

### Mutant *gltS* increases sensitivity to homocysteic acid

**Fig 3:**
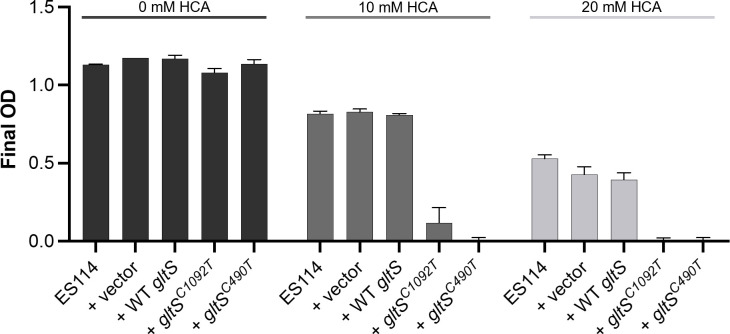
Expressing mutant GltS proteins increases sensitivity to HCA. Shown are final OD_600_ readings for *V. fischeri* cultures grown with increasing concentrations of HCA. Strains include ES114 alone, or carrying pVSV105, pMNC15 (*gltS*), pMNC16 (*gltS*^C1092T^), or pMNC17 (*gltS*^C490T^). Cultures were grown for 24 hours before reading OD_600_. Error bars indicate standard error of the mean (*n* = 4). Data from one representative experiment of at least three are shown.

In *Escherichia coli*, GltS acts as a relatively low-affinity transporter for homocysteic acid (HCA), a metabolite that inhibits growth (35, 36). Mutations in *gltS* can influence the sensitivity of *E. coli* to HCA (37), so we therefore tested the effect of *gltS* alleles on the sensitivity of *V. fischeri* to HCA by shuttling wild-type and mutant *gltS* alleles carried on pVSV105 into wild-type strain ES114. The mutant alleles conferred increased sensitivity to HCA, while the wild-type *gltS* and the parental shuttle vector did not (Fig. 3).

### Mutant *gltS* leads to altered PG structure

**Fig 4:**
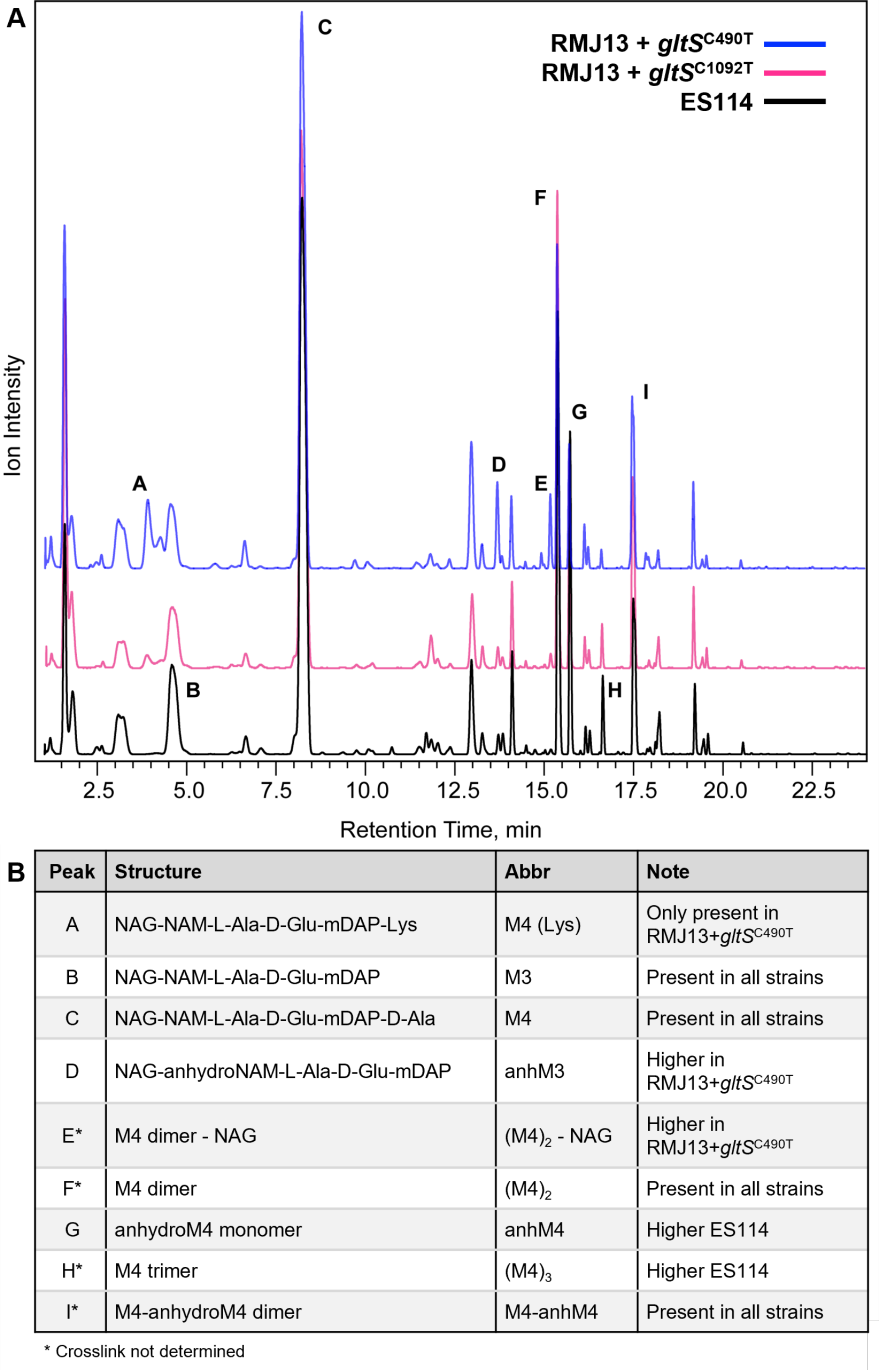
Muropeptide profiles of *V. fischeri* strains ES114, RMJ13 expressing *gltS*^C490T^ (carried on pMNC17), and RMJ13 expressing *gltS*^C1092T^ (carried on pMNC16). (A) Representative LC spectra from comparative muropeptide analysis in which the amount of purified and injected PG was normalized to the height of peak C. (B) Identification of peaks labeled in (A).

The wild-type PG structure in *V. fischeri*, and most other gram-negative bacteria, is composed of linear repeating strands of β-1,4 linked *N*-acetylglucosamine (NAG) and *N*-acetylmuramic acid (NAM), with short peptide chains covalently bound to most NAM molecules. The mature peptide chain bound to NAM is L-alanine, D-glutamate, *meso*-diaminopimelic acid (*m*DAP), and D-alanine. We hypothesized that RMJ13 expressing mutant *gltS* alleles would have wild-type PG structure, as the mutant GltS symporters appear to enable growth on small amounts of D-glu, rather than supporting D-glu-independent growth. Liquid chromatography-mass spectrometry (LC-MS) indicated that RMJ13 expressing *gltS*^C490T^ mostly contains PG indistinguishable from wild-type, with D-glu in its peptides (Fig. 4). However, LC-MS also showed another relatively minor peak corresponding to a molecule about 128 Da larger than wild-type monomers (Fig. 4). Further analysis revealed this subset of PG is composed of the wild-type NAG-NAM-tetrapeptide moiety with an additional D-lys bound to the fourth position D-ala (Fig. 4B; Fig. S1). The addition of D-lys in this position was only observed in the D-glu auxotroph expressing *gltS*^C490T^ present on pMNC17. PG from the same D-glu auxotroph carrying *gltS*^C1092T^ was not convincingly distinguishable from the PG of wild-type *V. fischeri* (Fig. 4A). Additionally, RMJ13+pMNC17 has different relative amounts of trimers and anhydrous monomers than the wild-type and RMJ13+pMNC16. The molecules corresponding to these peaks do not contain D-lys, so differences may be due to variance in cross-link, which was not determined for this study.

### *In silico* analysis of altered GltS

**Fig 5:**
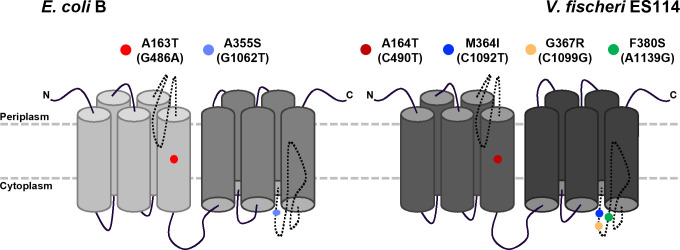
Diagram of predicted GltS protein structures of *E. coli* and *V. fischeri*. (Left) Structure of GltS of *E. coli* B strain WM335, which has two amino acid substitutions compared to wild-type. Both mutations (shown as colored dots) were found within the same strain. The diagram is based on SWISS-MODEL predictions and previous publications (42, 43, 45–48). (Right) Structure of GltS from *V. fischeri*, showing amino acid substitutions corresponding to mutations in RMJ13 suppressor strains. All mutations (shown as colored dots) are shown on one structure, although only one mutation exists per strain. Diagram based on SWISS-MODEL predictions (46–48).

The BLAST alignment tool (38–40) indicated that GltS from *V. fischeri* shares 48% identity and 64% similarity with GltS from *E. coli* B (EcGltS). Like EcGltS, GltS from *V. fischeri* is predicted to be an integral membrane protein in the cytoplasmic membrane (41–44). GltS from each of these bacteria is predicted to consist of two domains, each with five transmembrane helices and a pore loop that is likely involved in substrate specificity (42, 43, 45–48) (Fig. 5). The four mutant *gltS* alleles in our suppressor strains each have an amino acid substitution: A164T, M364I, G367R, and F380S, respectively (Fig. 5). Based on SWISS-MODEL predictions, one substitution, A164T, is found within a transmembrane helical region, while the other three are predicted to be clustered within a pore loop (Fig. 5) (46–48).

### RMJ13 colonizes *E. scolopes***,** dependent upon exogenous D-glu

**Fig 6:**
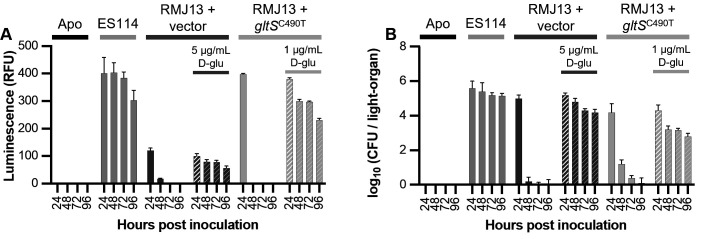
Colonization of *E. scolopes* by *V. fischeri* ES114 RMJ13-derived strains in the presence and absence of D-glu supplementation. (A) Squid luminescence over time for wild-type (ES114) and RMJ13 carrying pVSV105 (vector) or pMNC17 (*gltS*^C490T^), with and without persistent exogenous D-glu supplementation. “Apo” indicates uninoculated, aposymbiotic squid as negative controls. (B) Symbiont population levels (average CFU per light organ) over time, with the same strains, conditions, and treatments as in panel A. Error bars indicate standard error of the mean (*n* = 13). Data from one experiment are shown. This experiment was performed once.

**Fig 7:**
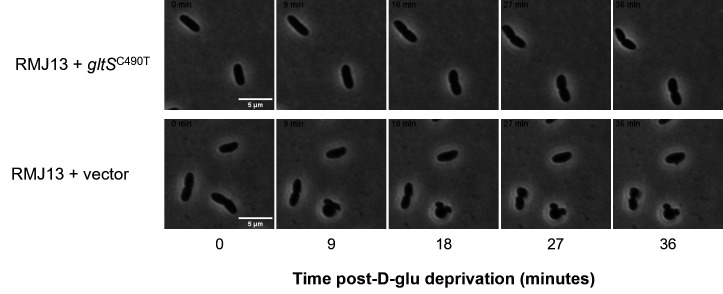
D-glu auxotrophic cells lyse when placed in filtered seawater and without D-glu. Cells lyse within 4 hours of being placed in filtered seawater without D-glutamate. Expression of *gltS*^C490T^ (top) does not have a growth advantage over RMJ13 carrying the vector alone (bottom) in these conditions.

Due to the importance of PG as a signaling molecule in the squid-*Vibrio* symbiosis, we sought to determine whether the D-lys addition to PG caused by *gltS*^C490T^ on pMNC17 affected symbiotic interactions. We therefore inoculated hatchlings with RMJ13 carrying *gltS*^C490T^ and a parental vector control. RMJ13 with the empty vector (pVSV105), as well as RMJ13 expressing *gltS*^C490T^ (pMNC17), was able to colonize hatchling *E. scolopes* (Fig. 6). Hatchling squid were inoculated overnight in filtered seawater (FSW) containing *V. fischeri*, with both RMJ13-derived strains also receiving supplementation with D-glu. Squid were then transferred into fresh FSW; half of each RMJ13-inoculated group was transferred to FSW with D-glu, while the others were placed in FSW without D-glu, to determine if lack of D-glu in FSW interrupted colonization by D-glu auxotrophs. Viability of the symbionts was measured by average CFU per light organ and luminescence (Fig. 6). Both RMJ13 carrying the empty vector (pVSV105) and RMJ13 expressing *gltS*^C490T^ (pMNC17) were able to colonize the squid when D-glu was present, but luminescence of squid infected with either strain rapidly decreased after being transferred into seawater lacking D-glu (Fig. 6A). Additionally, the average CFU per light organ of squid infected with each RMJ13 strain decreased rapidly after depriving them of D-glu, aligning with the luminescence data (Fig. 6B). These results parallel the lysis of D-glu auxotrophic strains when D-glu is no longer supplemented in seawater (Fig. 7).

## DISCUSSION

While it was established long ago that L-amino acids are vitally important building blocks of proteins in all life forms, there has been growing appreciation and study of D-amino acids more recently. Though they are less commonly found in nature, D-amino acids do have fundamental roles in most organisms and ecosystems (1, 2, 5, 7–9, 49, 50). For example, D-amino acids are constituents of the PG peptide in bacterial cell walls (51). In most gram-negative bacteria, PG includes D-glu, *m*DAP, and D-ala. In *V. fischeri*, each of these amino acids is primarily produced for PG biosynthesis by a specific enzyme. D-ala is produced by Alr (24), *m*DAP is likely produced by a putative DAP epimerase encoded by the *dapE* gene (30, 31), and the D-glu needed for PG biosynthesis is mainly produced by MurI (24). In *V. fischeri*, RacD (29) and BsrF (16) can also provide D-glu for PG, though they do not appear to contribute significantly to PG biosynthesis under normal growth conditions in a wild-type background.

Previous studies sought to alter *V. fischeri*’s PG biosynthesis by creating strains auxotrophic for PG-specific D-amino acids and then selecting for prototrophic suppressors (16, 29, 52). Most recently, we found that removal of a putative N-terminal secretion signal from the broad-spectrum racemase BsrF allowed the enzyme to produce sufficient cytoplasmic D-glu to suppress auxotrophy (16). Although ultimately the suppressor mutant grew on unsupplemented LBS, it was isolated on LBS medium supplemented with iso-D-glutamine. In the present study, we attempted to use exogenous D-gln to isolate suppressors of D-glu auxotrophy. In total, 10 suppressors were isolated, eight of which consistently required supplementation to grow in LBS-Em (Fig. 1). All 10 of these suppressors have a mutation in *gltS*, which encodes a putative sodium:glutamate symporter (30, 31) (Table 1). Four mutant *gltS* alleles were isolated, two of which arose independently in multiple cultures.

Other mutations deviating from wild type were also discovered in the suppressor mutants, including VF_0468^A699G^ and *gacS*^G644C^, which were each recovered independently from two mutants (Table 1). GacS is a sensor kinase and part of the GacS/GacA two-component regulatory system, which has a broad regulon and is widespread among many Proteobacteria, including *V. fischeri* (30, 31, 53, 54). VF_0468 appears to encode an ortholog of LspA, a prolipoprotein signal peptidase II that in other bacteria plays a role in cell envelope and PG synthesis. Given that neither of these mutations was isolated in the absence of a *gltS* mutation, it is uncertain whether they were primary mutations that contribute to survival in D-glu limiting conditions or secondary mutations that arose to compensate for negative effects associated with the *gltS* mutations themselves. VF_0468 was categorized as an essential gene (28), and the VF_0468^A699G^ allele may be of interest for future studies. In this study, we chose to focus on *gltS*, which was the only gene consistently mutated in the suppressors.

A well-studied homolog of *V. fischeri*’s GltS is that of *E. coli* (27, 36, 42, 43, 55–59). Of particular interest in comparison to this study, a D-glu auxotroph of *E. coli* could not be generated without concomitant mutations in *gltS*, presumably to enable transport of exogenously supplied D-glu (26, 27). Interestingly, the two mutations in *gltS* that allowed for generation of a D-glu auxotrophic strain of *E. coli* correspond to two of the mutant alleles generated in this study. The first of these, codon 163 in EcGltS and 164 in *V. fischeri* (allele *gltS*^C490T^), substitutes a threonine residue for alanine within the fifth transmembrane helix of the protein (Fig. 5) (27). The second allele, *gltS*^C1092T^ in the present study, is located at codon 355 in EcGltS and 364 in *V. fischeri* (27) (Fig. 5). Although neither the wild-type (methionine in *V. fischeri* and alanine in *E. coli*) nor substituted amino acids (isoleucine and serine) are similar to each other, it is intriguing that similarly aligned codons were found in a similar context of selective pressure to grow on exogenous D-glu. Three of the *gltS* mutations in this study are in a predicted pore loop (Fig. 5B) (46–48). Pore loops are often selectivity filters of transport proteins (60–64). We speculate that these mutations affect the GltS symporters to render them more permissive to the uptake of D-glu and generally more promiscuous, allowing uptake of HCA.

Because suppressors were selected on LBS-Em supplemented with D-gln, we initially hypothesized that altered GltS symporters were enabling mutants to access D-gln in the media, with D-gln either directly incorporated into PG or being metabolized to D-glu to build PG. However, when we determined that overexpression of mutant GltS supported growth of a D-glu auxotroph in unsupplemented LBS (Fig. 2B), we instead speculated that the components of rich media may contain low amounts of D-glu that are sufficient to support growth if they can be accessed by enhanced D-glu transport. Our results support this possibility, as we found approximately 1.4 µM D-glu in unsupplemented LBS (Table 2), which is sufficient to support growth of the D-glu auxotroph expressing either GltS mutant in chemically defined FMM (Fig. 2B). Moreover, based on estimates that there are 3.5 million PG monomers per cell in *E. coli* (65), even this low concentration of D-glu is theoretically more than enough to achieve a turbid culture of more than 10^8^ cells per milliliter, if D-glu in the medium is accessible and able to be incorporated into PG.

Our results are consistent with a model whereby our *gltS* mutants, and their amplification, were enriched under selective pressure to access small amounts of D-glu. Our data imply that the D-glu in LBS is provided by the yeast extract (Table 2). Although heat may catalyze abiotic racemization, similar amounts of D-glu were seen in media that have been autoclaved and filter-sterilized, suggesting that D-glu is either provided directly from the yeast used to produce yeast extract or is abiotically racemized from L-glu during production or storage of yeast extract (1, 66). Additionally, our stock solution of D-gln appears to contain enough D-glu that, when added into LBS, sufficient D-glu was added for the original suppressor mutants to grow, even with mutant *gltS* alleles in single copy, though the concentration is too low to sustain growth of the parental D-glu auxotroph (Fig. 1B; Table 2). Spontaneous deamidation of glutamine has been previously studied (32, 67–71). It was found that glutamine is relatively unstable, degrading to ammonia and glutamate. Though these studies have mainly focused on the L-form, it seems likely that a similar process could have occurred with our D-gln stock at standard lab conditions. Such deamidation would account for both the lower-than-expected amount of D-gln and the relative concentration of D-glu in our D-gln stock. Taken together, our results suggest that yeast extract, and thereby LB and other rich complex media, contain trace amounts of some D-amino acids, including D-glu. This knowledge could lend itself to selecting for strains or proteins with increased capacity to transport D-amino acids.

An important drawback to the increased efficiency of mutant GltS proteins in both *V. fischeri* and *E. coli* is the increased sensitivity to toxic glutamate analogs, such as HCA (Fig. 3), and even sensitivity to D-glu itself (35–37, 59). HCA inhibits bacterial growth by competitively binding to MurD (72), the enzyme that adds D-glu to the growing PG peptide (11). D-glu itself can also have an inhibitory effect at high enough concentration by affecting enzymes of ammonia assimilation in *Bacillus megaterium*, leading to altered growth phenotypes (73, 74). These data illustrate a fitness trade-off: although such strains can scavenge trace amounts of D-glu from their surroundings to build wild-type PG, they become more sensitive to toxic HCA or high environmental D-glu. This conclusion adds appeal to the use of glutamate analogs as antimicrobial drugs against bacteria with sufficient glutamate transport activity.

An important finding in this project is the variability of PG structure: our data show that RMJ13 expressing *gltS*^C490T^ has a subset of PG peptides with D-lys in the fifth amino acid position. D-lys previously has been found in PG at the third position in *Thermotoga maritima* (75) and at the terminal fourth or fifth position in *Acinetobacter baumannii* (76). In the latter case, D-lys is produced by the racemase RacK, which contains a signal sequence for secretion to the periplasm (76), where incorporation of D-lys into PG is thought to occur through the activity of transpeptidase(s) and penicillin-binding proteins (12, 77–79). Based upon its specificity for glutamate homologs, it seemed unlikely that GltS itself was responsible for D-lys incorporation in PG. Instead, we hypothesize that D-lys was added to the mature PG peptides due to the activity of the periplasmic broad-spectrum racemase BsrF in a manner similar to that proposed in *A. baumannii* (76). The most well-characterized homolog of BsrF is *Vibrio cholerae*’s BsrV, which produces non-canonical D-amino acids that can be added to mature PG, possibly to promote resistance to various environmental stressors (6). In the current study, BsrF may be producing D-lys, which subsequently is added to mature PG peptides to combat cell wall stress. This possibility could be tested by expressing *gltS*^C490T^ in a D-glu auxotroph that no longer produces BsrF.

Despite its altered PG peptides, RMJ13 expressing *gltS*^C490T^ is able to colonize *E. scolopes* to the same level as the wild-type, so long as there is D-glu in the seawater (Fig. 6). In this context, the expression of *gltS*^C490T^ does not provide any apparent advantages over the empty vector. As seen in Fig. 7, cells lyse within 4 hours of being suspended in seawater lacking D-glu, due to insufficient PG biosynthesis and/or remodeling. These results mirror those of our earlier work with D-ala auxotrophs (34) and add strength to our assertion that D-amino acid auxotrophs are viable candidates for studying transient colonization of the Hawaiian bobtail squid. More work can be done to elucidate whether the altered PG peptide itself has any effect on squid colonization. As shown in Fig. 4A, only a small subset of the PG has this extra lysine, while a majority has the wild-type structure, so the wild-type PG signal the squid typically receives (80) is still present and presumably predominant. The best way to observe specific effects of the altered peptide would be to isolate the altered-monomer peak from LC-MS experiments, expose juveniles directly to the molecule, and test the effects on the host’s morphogenic programs that are normally induced by PG (15, 81–83). Such experiments would inform our understanding of the structure-function relationship between PG and symbiotic signaling.

## MATERIALS AND METHODS

### Bacterial strains and culture conditions

The strains used in this study are listed in Table 3. When added to LB medium (84) for selection of *E. coli*, chloramphenicol (Cm) and kanamycin were used at concentrations of 20 and 40 µg/mL, respectively. When added to LBS (85), FMM minimal medium (16, 34), and FSW for selection of *V. fischeri*, Cm and Em were used at concentrations of 2 and 5 µg/mL respectively. Agar was added to a final concentration of 1.5% for solid media. LBS, TBS (20 mM Tris-hydrochloride [Tris] (pH 7.5), 10 g/L tryptone, and 20 g/L NaCl), and YEBS (20 mM Tris [pH 7.5], 5 g/L yeast extract, and 20 g/L NaCl) were autoclaved before use. Stock solutions of D-glu (99+% powder, Sigma Aldrich, St. Louis, MO) and D-gln (99+% powder, Thermo Fisher Scientific Inc., Waltham, MA) were prepared by dissolving 40 mg/mL of powder in deionized water. NaOH was added to 250 mM to dissolve D-glu. D-gln was incubated at 37°C, shaking until completely dissolved. Stock solutions were filter-sterilized with VWR sterile syringe filters (25 mm 0.22 µm; VWR, Radnor, PA) attached to BD Luer-Lok tip syringes (Becton, Dickinson and Company, Franklin Lakes, NJ) and were stored at room temperature. HCA was dissolved in water to create a stock solution of 250 mM (45.8 mg/mL), filter-sterilized, and stored at 4°C.

**TABLE 3:** Strains and plasmids used in this study^*a*^^,^^*b*^

Strain	Genotype	Source
*E. coli*		
DH5α	φ80d*lacZ*ΔM15 ∆(*lacZYA-argF*)U169 *deoR supE44 hsdR17 recA1 endA1 gyrA96 thi-1 relA1*	(86)
DH5αλ*pir*	DH5α lysogenized with λpir	(87)
CC118λ*pir*	*∆*(*ara-leu*)*araD ∆lac74 galE galK phoA20 thi-1 rpsE rpsB argE*(Am) *recA* λ*pir*	(85)
*V. fischeri*		
AKD100	ES114 with a miniTn*7*-Em	(88)
ES114	Wild-type isolate from *E. scolopes*	(89)
RMJ13	*murI*::miniTn*5*-Em ∆*racD*	(29)
RMJ13.M1	*murI*::miniTn*5*-Em ∆*racD gltS*^C1099G^	This study
RMJ13.M2	*murI*::miniTn*5*-Em ∆*racD gltS*^C490T^ *gacS*^G644C^	This study
RMJ13.M3	*murI*::miniTn*5*-Em ∆*racD gltS*^C1092T^; new duplication junction including VF_A0495 to VF_A0526 [ACTTAACTTGAT::GATGTTGTTTTA]	This study
RMJ13.M3.1	*murI*::miniTn*5*-Em ∆*racD gltS*^C1092T^ VF_2147^G778A^ amplification junction including VF_A0495 to VF_A0526 [ACTTAACTTGAT::GATGTTGTTTTA]	This study
RMJ13.M4	*murI*::miniTn*5*-Em ∆*racD gltS*^C490T^ *gacS*^G644C^	This study
RMJ13.M5	*murI*::miniTn*5*-Em ∆*racD gltS*^C1139G^	This study
RMJ13.M6	*murI*::miniTn*5*-Em ∆*racD gltS*^C1099G^ VF_0648^A699G^	This study
RMJ13.M8	*murI*::miniTn*5*-Em ∆*racD gltS*^C490T^ new duplication junction including VF_A0498 to VF_A0571 [TGTGCTGATAAA::AGGTGAAAAGGG]	This study

Plasmid	Relevant Characteristics	Source
pCR-Blunt II-TOPO	*ori*V_ColE1_, *kanR*	Thermo Fisher
pEVS104	Conjugative helper plasmid; *oriV*_R6K_ *oriT*_RP4_ *kanR*	(85)
pMNC11	Wild-type *gltS* from ES114 cloned into pCR-Blunt II-TOPO	This study
pMNC12	*gltS*^C1092T^ from RMJ13M3.1 cloned into pCR-Blunt II-TOPO	This study
pMNC13	*gltS*^C490T^ RMJ13M2 cloned into pCR-Blunt II-TOPO	This study
pMNC15	Wild-type *gltS* cloned into pVSV105	This study
pMNC16	*gltS*^C1092T^ cloned into pVSV105	This study
pMNC17	*gltS*^C490T^ cloned into pVSV105	This study
pMNC28	*gltS*^C1099G^ from RMJ13M7 cloned into pCR-Blunt II-TOPO	This study
pMNC29	*gltS*^C490T^ from RMJ13M9 cloned into pCR-Blunt II-TOPO	This study
pVSV105	*oriV*_R6K_, *oriV*_pES213_, *oriT*_RP4_, *cmR*, *lacZα*	(33)

Primer^*c*^	Sequence	Source
MNC28	CAT CCT AGG ACT AGT GAT ATT TCA ACT TAG GAG TAC TAT G	This study
MNC29	CAT CCT AGG TAT GAG AGG TAG GGC TTT TTT CTT A	This study

^*a*^ Drug resistance abbreviation used: Em, erythromycin resistance.

^*b*^ Alleles cloned in this study are from *V. fischeri* strain ES114. Replication origins (oriV) on each vector are listed as RR6Kγ, ColE1, and/or pES213. Plasmids based on pES213 are stable in *V. fischeri* and do not require antibiotic selection for maintenance (33).

^*c*^ Oligonucleotides are shown in the 5´-to-3´ direction. Underlined regions are restriction-enzyme recognition sites.

### Molecular genetics and sequence analysis

Oligonucleotides used for PCR and cloning are listed in Table 3, and were synthesized by Integrated DNA Technologies (Coralville, IA). DNA ligase and restriction enzymes were purchased from New England Biolabs (Beverly, MA). PCR was conducted with Phusion DNA polymerase (New England Biolabs). Plasmids used for cloning were prepared with the ZymoPURE Plasmid Miniprep Kit (Zymo Research, Irvine, CA). DNA was cleaned after PCR and between cloning steps using the DNA Clean & Concentrator Kit from Zymo Research. Cloned plasmids were Sanger sequenced at the University of Illinois-Chicago Genome Research Core facility and analyzed via Geneious Prime version 2019.0.4. Genomic DNA from *V. fischeri* strains was extracted using the Invitrogen PureLink Genomic DNA Mini Kit (Thermo Fisher Scientific Inc.). For whole-genome sequencing, DNA was sonicated to approximately 500 bp fragments, then DNA libraries were prepared using the NAGNext Ultra II DNA library prep kit for Illumina (New England Biolabs), including end-repair, adaptor ligation, and addition of index primers. Libraries were sequenced at the University of Georgia Genomics and Bioinformatics Core (Athens, GA). All sequences were analyzed via Geneious Prime with default settings, compared to *V. fischeri* wild-type strain ES114. Paired-end reads were mapped to the reference, then single-nucleotide polymorphisms were identified with a minimum variant frequency of 0.8.

### Plasmid construction

Plasmids used in this study are listed in Table 3. Plasmids were maintained in *E. coli* DH5α, with the exception of pVSV105 and its derivatives, which were maintained in DH5αλpir*,* and pEVS104, which was maintained in CC118λ*pir* (85). When relevant, plasmids were conjugated into *V. fischeri* via triparental mating with helper plasmid pEVS104. Complementation plasmids pMNC15, pMNC16, and pMNC17 were produced by PCR amplifying *gltS* from ES114, RMJ13M3.1, and RMJ13M2, respectively, using primers MNC28 and MNC29. PCR products were then cloned into pCR-Blunt II TOPO (Thermo Fisher Scientific Inc.), yielding pMNC11, pMNC12, and pMNC13, respectively. These plasmids were then digested with AvrII, and the *gltS*-containing fragments were ligated into XbaI-cut pVSV105, producing pMNC15, pMNC16, and pMNC17, respectively. Plasmids used for targeted gene sequencing of *gltS* in RMJ13S7 and RMJ13S9 were produced by PCR amplifying *gltS* with primers MNC28 and MNC29, then cloning the products into pCR-Blunt II TOPO.

### Selection of spontaneous mutants of D-glu auxotrophy

Strain RMJ13 was grown in LBS-Em containing 400 µg/mL D-glu to an OD_600_ of 1. A total of 100 µL of culture was plated to LBS-Em supplemented with 400 µg/mL D-gln. Cultures were plated in parallel on D-glu plates to determine the number of CFU. Plates were incubated at 28°C. CFU counts from D-glu-supplemented plates were counted at 24 hours, while suppressor colonies on D-gln-containing plates were counted at 48 hours post-inoculation. Suppressor colonies were streak purified on LBS containing Em and D-gln, then stocked in LBS with 20% glycerol and stored at −80°C.

### Analysis of amino acid content in media and amino acid solutions

Media samples and amino acid stock solutions were analyzed at the University of Illinois Chicago Mass Spectrometry Core. Amino acids were diluted into 4 mg/mL working solutions and, along with samples of LBS, TBS, and YEBS, were filter-sterilized with VWR sterile syringe filters (25 mm diameter, 0.22 µm pore size; VWR) attached to BD Luer-Lok tip syringes (Becton, Dickinson and Company) before submission. Three 1 mL samples of each solution were submitted to the Mass Spectrometry Core in the Research Resources Center of the University of Illinois Chicago for analysis of L-/D-glu and L-/D-gln concentrations. LC-MS-grade analytes were purchased from Sigma-Aldrich (Burlington, MA) and dissolved in water to get stock solutions of 1 mg/mL. They were diluted in LC-MS-grade 50% MeOH in water to create spiking standards to prepare standard curves. Stable isotope-labeled amino acid mix solution (Millipore Sigma, Burlington, MA) was diluted with 50% MeOH to create a 1 µg/mL working solution, used as the internal standard. Solid phase extraction was done using Oasis MCX Cartridge. LC-MS analyses were performed on an AB SCIEX 6500 QTRAP coupled with Agilent 1290 UPLC system, with an Agilent Poroshell 120 Chiral-T, 2.7 µm, 2.1 × 100 mm column. Data analysis was conducted by Sciex MultiQant software (version 3.0.3, AB Sciex Pte, Ltd., Burmingham, MA).

### Peptidoglycan isolation from intact PG sacculi

Cells were grown overnight in LBS with any necessary antibiotics and amino acids, chilled on ice for 10 min, and centrifuged at 4°C and 17,600 × *g* for 15 min. To prevent precipitation at later steps, pellets of *gltS* mutant strains and strains carrying pMNC16 or pMNC17 were washed by resuspension in 400 mL of 1 M NaCl and centrifuged as above. Pellets were resuspended in 10 mL cold water, then dripped into 50 mL of boiling 4% SDS. The solution was then boiled for 30 min with continuous stirring and allowed to cool to room temperature. Samples were then centrifuged at 120,000 × *g* for 60 min, resuspended in room temperature water, and washed three to four more times as above. Before resuspension, the supernatant was assayed for SDS using methylene blue and chloroform (90) and centrifuged as above until no SDS was detected in the supernatant. When SDS was undetectable, the pellet was resuspended in 1 mL of 1 M Tris-HCl (pH 7.5) in water, then treated with 10 µg DNase I and 50 µg RNase A for 30 min at 37°C. Samples were then treated with 100 µg of trypsin, and CaCl_2_ was added to a final concentration of 10 mM and incubated overnight at 37°C. Samples were then centrifuged at 15,880 × *g* for 10 min, and pellets were resuspended in 1% SDS. The solution was incubated in a 95°C hot water bath for 20 min, diluted with warm water, and then centrifuged at 120,000 × *g* for 60 min at room temperature. The pellet was then washed with warm water and repeatedly centrifuged and pelleted as above until SDS-free. The pellet was resuspended in 1 mL water and stored at −80°C until analysis or further processing as below.

### Peptidoglycan processing for amino acid and muropeptide analysis

Intact PG sacculi were resuspended in 12.5 mM NaPO_4_ (pH 5.5). Samples were digested with 125 units of mutanolysin overnight at 37°C. Insoluble material was then removed from the samples by centrifugation at 15,880 × *g* for 15 min at room temperature. The supernatant containing muropeptides was transferred to a new tube, lyophilized until dry, then stored at −20°C until analysis.

### LC-MS analysis of PG samples and data analytics

Muropeptide and sacculi structural analyses were performed on a Shimadzu LCMS-9030 QToF instrument interfaced with an LC-40B X3 UPLC, a SIL-40C X3 autosampler (10°C), and a CTO-40C column oven (40°C). Gradient separations utilized a BEH C18 column (2.1 mm × 50 mm, 1.7 µm particle size; Waters) with solvent A (0.1% formic acid in water) and solvent B (0.1% formic acid in MeOH) at a constant flow rate of 0.4 mL min^−1^. Experiments were performed and analyzed as previously described (91).

### Squid colonization assay

*V. fischeri* ES114 was cultured overnight at 28°C in LBS medium, and RMJ13 strains carrying pVSV105 or pMNC17 were grown in LBS with 50 μg/mL of D-glu, 5 μg/mL Em for retention of the mini-Tn*5,* and 2 µg/mL Cm for plasmid retention. Overnight cultures were diluted 100-fold into seawater tryptone liquid medium with D-glu and antibiotics as necessary and allowed to grow until mid-exponential phase at 28°C, then diluted to a final inoculum concentration of ~5,000 CFU/mL in 100 mL of filter-sterilized ocean water (FSW). Inoculum of RMJ13 carrying pVSV105 was supplemented with 5 µg/mL D-glu, while inoculum of RMJ13 carrying pMNC17 received 1 µg/mL D-glu (strains were given different concentrations based on their needs for growth). Newly hatched *E. scolopes* were introduced into this mixture and inoculated overnight under a 12/12 day-night cycle. After about 16 hours, squid were transferred into individual vials: ES114-inoculated squid were transferred into FSW, while half of each RMJ13-inoculated cohort was transferred into either FSW or FSW with D-glu. At 24 hours post-inoculation and every 24 hours following, luminescence was measured using a TD 20/20 luminometer (Turner Designs, Sunnyvale, CA), after which the squid were either transferred to fresh FSW with D-glu as needed or frozen at −80°C in 700 µL of FSW until plated or dissected. Most squid were individually homogenized, then dilution plated to LBS agar medium, and the number of CFU/mL was determined.

### Microscopy of D-glu auxotrophic bacteria

RMJ13 carrying the empty vector (pVSV105) or *gltS*^C490T^ (pMNC17) was initially grown in LBS containing Em and Cm, and the RMJ13 carrying pVSV105 was supplemented with 50 µg/mL D-glu. When cultures reached an OD_600_ of ~0.6, cells were harvested by centrifugation at 7,000 × *g* for 1 min at room temperature, washed with FSW, and then resuspended in FSW without D-glu supplementation. Every hour, aliquots were taken from the cultures, and fixed with ice-cold 70% ethanol in water, then incubated on ice for 1 hour. Cells were centrifuged at 10,000 × *g* at room temperature for 1 min, then resuspended in FSW. A 1.5% agarose pad was made in FSW with SeaKem LE agarose (Lonza Biosciences, Morristown, NJ). A total of 1 µL aliquot of cell sample was loaded onto a coverslip, and the agarose pad was placed on top. Images were captured by an inverted Zeiss LSM 980 microscope equipped with a Plan Apochromat 1.4NA 100× oil phase 3 objective at the Caltech Biological Imaging Facility. Images were processed and visualized in ImageJ.

## Data Availability

Illumina reads from whole-genome sequencing of ES114 and RMJ13 suppressors are available in NCBI’s Sequence Read Archive (SRA) under accession numbers SRX23390107 (ES114) and SRX23390108 through SRX23390115 (RMJ13 suppressor strains), and all other raw and derived data supporting the findings of this study are available from the corresponding author E.V.S. upon request.

## SUPPLEMENTAL MATERIAL

Fig. S1 spectrum.01025-25-s0001.pdf Amino acid D/L determination using Marfey's Reagent.

ASM does not own the copyrights to Supplemental Material that may be linked to, or accessed through, an article. The authors have granted ASM a non-exclusive, world-wide license to publish the Supplemental Material files. Please contact the corresponding author directly for reuse.
