# Supplementary material for: Mutant gltS alleles enable a Vibrio fischeri D-glutamate auxotroph to grow with lower requirements for exogenous D-glutamate
Source: Microbiol Spectr. 2025 Sep 4;13(10):e01025-25. doi: 10.1128/spectrum.01025-25 (PMC12502697; doi:10.1128/spectrum.01025-25)
Supplement: Fig. S1 — Amino acid D/L determination using Marfey's Reagent. [file spectrum.01025-25-s0001.pdf]

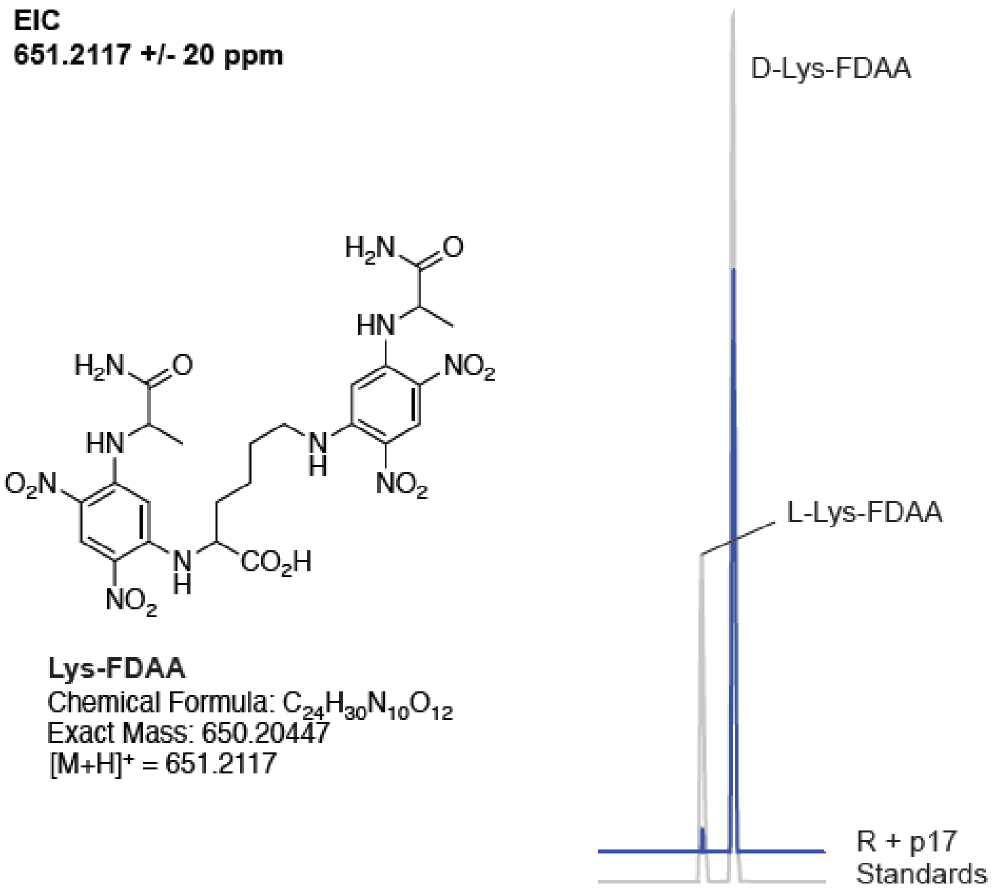

Figure S1. Amino acid *D/L* determination using Marfey's Reagent.

Muropeptides were acid hydrolyzed in 6N HCl in vacuum hydrolysis tubes as recommended (ThermoFisher, Cat. No. 29570, MAN0011742. The derivatized amino acids were analyzed by LC-MS using a Shimadzu 9030 QtoF interfaced with a LC-40B X3 UPLC, a SIL-40C X3 autosampler (10°C) and a CTO-40C column oven (40°C).
